# Supplementary material for: Autophagy-mediated degradation of integumentary tapetum is critical for embryo pattern formation
Source: Nat Commun. 2024 Mar 27;15:2676. doi: 10.1038/s41467-024-46902-8 (PMC10973531; doi:10.1038/s41467-024-46902-8)
Supplement: Supplementary file 1 — Supplementary Information [file 41467_2024_46902_MOESM1_ESM.pdf]

Autophagy-Mediated Degradation of Integumentary Tapetum Is Critical for Embryo Pattern Formation

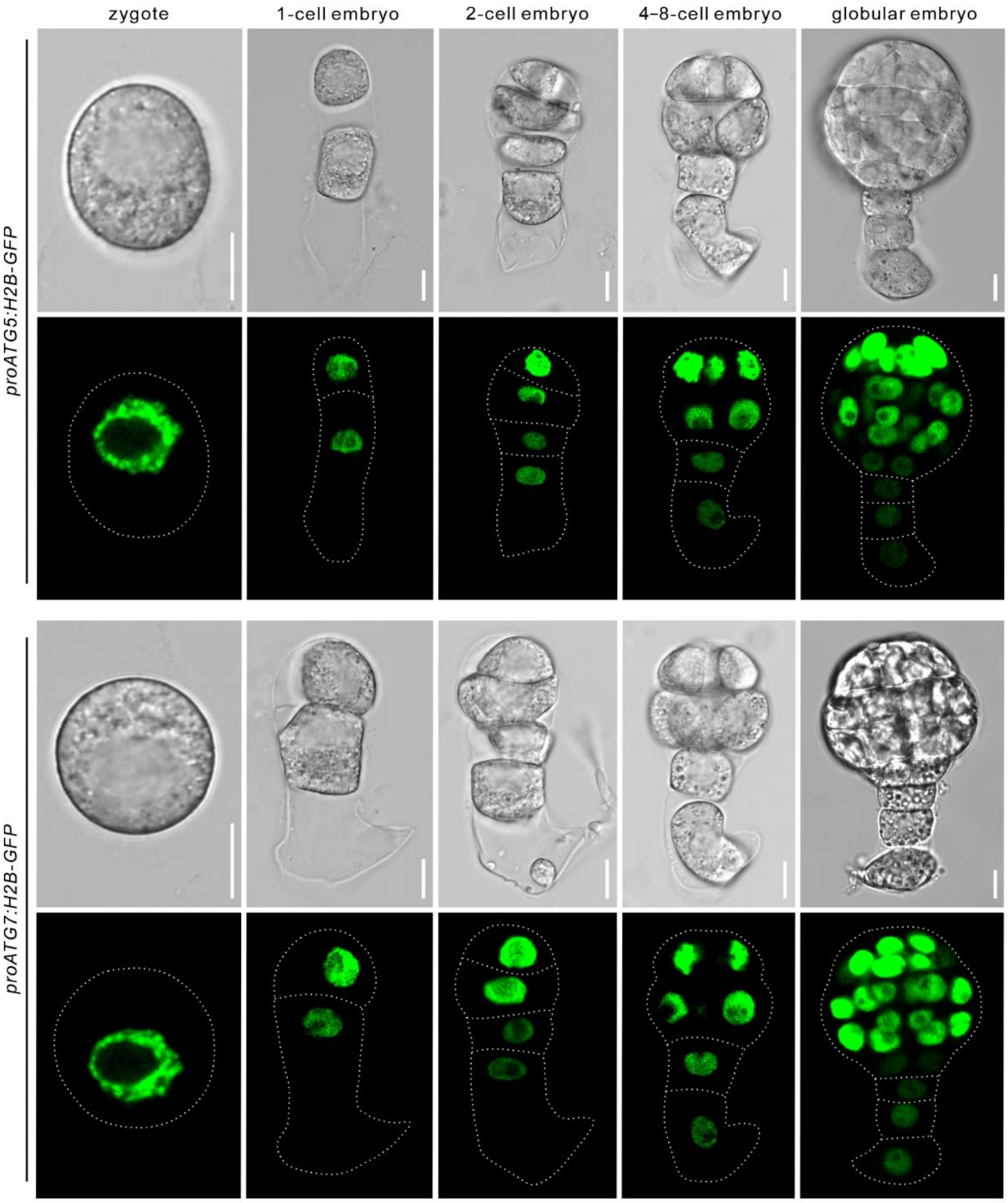

Supplementary Fig. 1 *ATG5* and *ATG7* are expressed in early embryos.

Analysis of *proATG5:H2B-GFP* and *proATG7:H2B-GFP* reporter lines revealed that both *ATG5* and *ATG7* are expressed in zygotes and early embryos. Scale bars: 10 μm. Observation of GFP in *proATG5:H2B-GFP* and *proATG7:H2B-GFP* reporter lines was repeated two times with similar results.

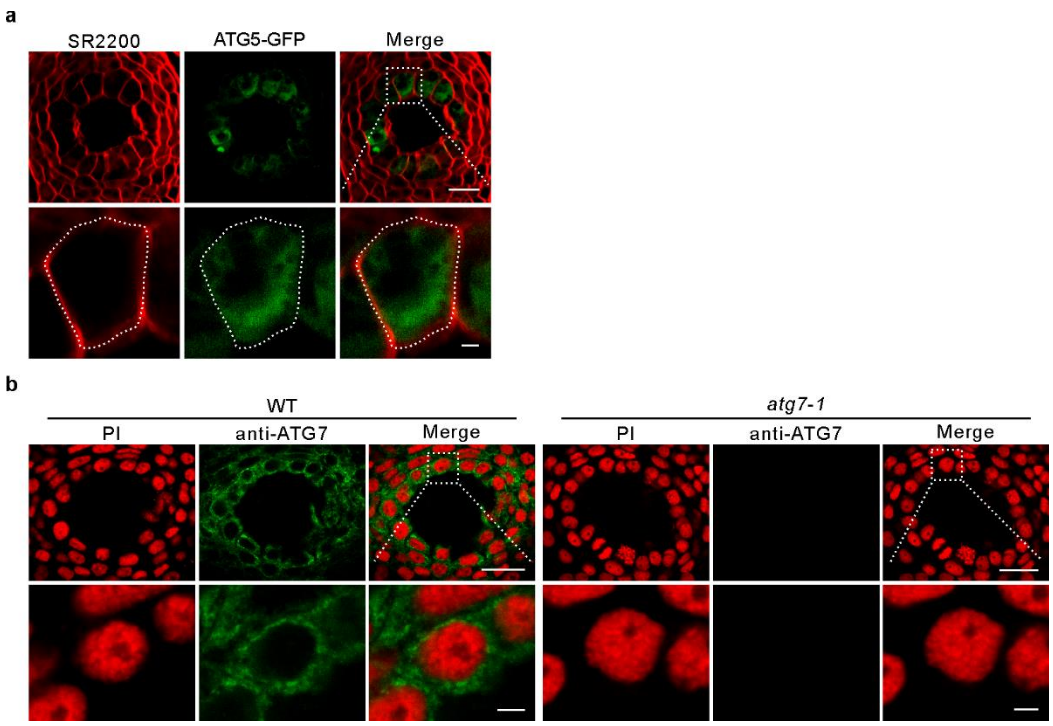

Supplementary Fig. 2 Subcellular localization analysis of ATG5 and ATG7 in the integumentary tapetum.

a Assessing the subcellular localization of ATG5 in the integumentary tapetum by examining ATG5-GFP fluorescence from the *proTPE8:ATG5-GFP* transgenic plants. The lower row shows a magnified view of the integumentary tapetal cell outlined by the dotted lines. SR2200, SCRI Renaissance 2200. Scale bars: 20 μm (upper row); 2.5 μm (lower row). **b** Analysis of the ATG7 protein subcellular localization in the integumentary tapetum by ATG7 immunofluorescence. The *atg7-1* mutant was used as the negative control. The lower row is the magnified view of the integumentary tapetal cells outlined by the dotted lines. PI, propidium iodide; WT, wild type. Scale bars: 20 μm (upper row); 2.5 μm (lower row). Subcellular localization analysis experiments were repeated three times with similar results.

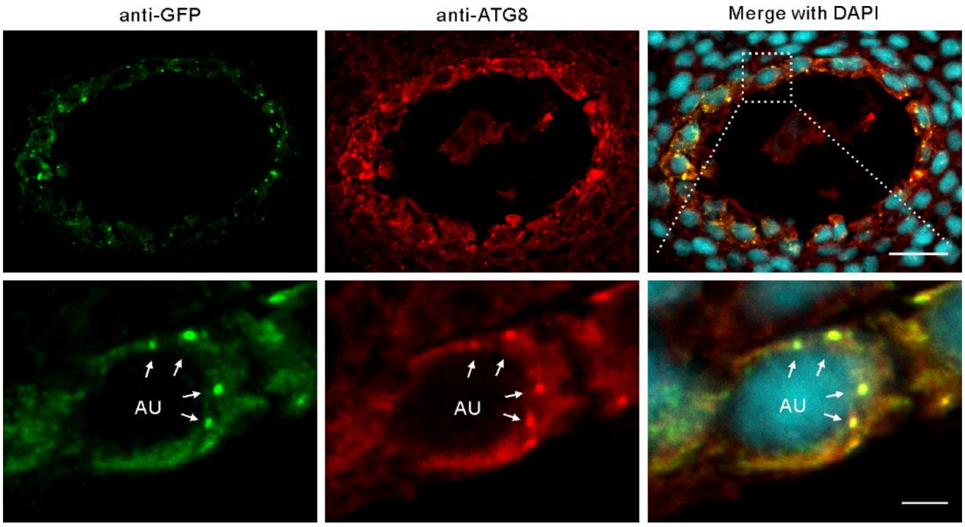

Supplementary Fig. 3 Double immunofluorescence visualizes autophagosomes in the integumentary tapetum.

Double immunofluorescence staining revealed co-localization of GFP-labeled and ATG8-labeled autophagosomes in the integumentary tapetum expressing *proTPE8:GFP-ATG8*. Nuclei were stained with 4', 6-diamidino-2-phenylindole (DAPI). The lower row is the magnified view of the integumentary tapetal cell outlined by the dotted lines. AU, autophagosomes. Scale bars: 20 μm (upper row); 2.5 μm (lower row). Double immunofluorescence experiment was repeated three times with similar results.

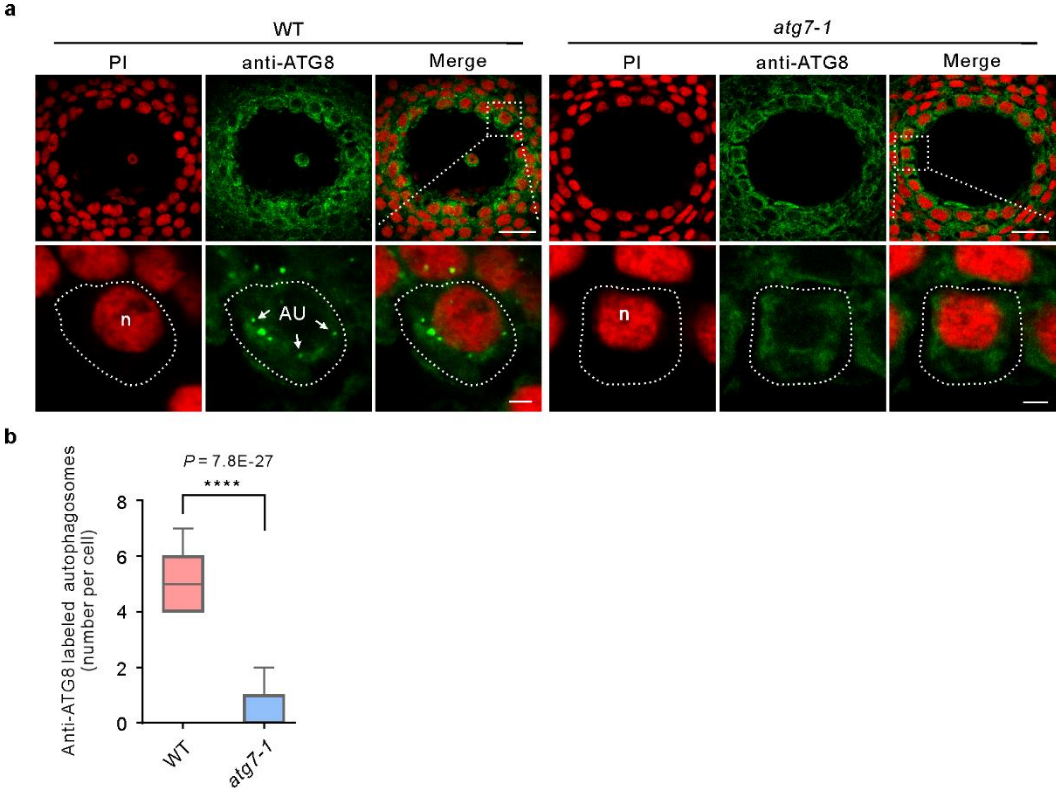

Supplementary Fig. 4 Reduced autophagic activity in the integumentary tapetum of *atg7-1* mutant.

a Autophagosomes in the integumentary tapetum of both the wild type (WT) and *atg7-1* mutant were visualized through ATG8 immunofluorescence. The lower panel presents an enlarged view of an integumentary tapetal cell delineated by dotted lines. Arrows indicate autophagosomes. PI, propidium iodide; n, nucleus. AU, autophagosomes. Scale bars: 20 μm (upper row) and 2.5 μm (lower row). **b** Quantification of autophagosomes in the integumentary tapetum of the WT and *atg7-1* mutant**.** The center line indicates the median value, while the top and bottom of the boxes represent the 75th and 25th percentiles, respectively. This description pertains to all subsequent box-and-whisker plots in the supplementary information. Data are from three independent experiments, each experiment with 10 independent samples (n = 30). (Two-tailed Student´s *t* test, $^{****}P < 0.0001$).

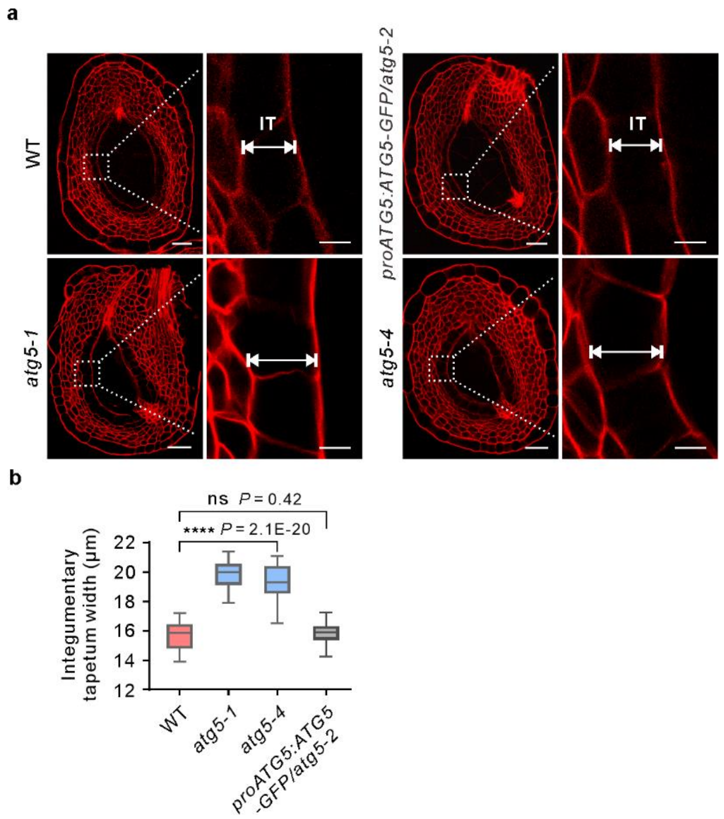

Supplementary Fig. 5 Mutation in *ATG5* leads to defects in integumentary tapetum degradation.

a Modified pseudo-Schiff-propidium iodide (mPS-PI) staining reveals seeds at 4 days after pollination (DAP) from the WT, *atg5-1*, *atg5*-4 and *proATG5:ATG5-GFP* complementation plants. The images on the right show the magnified area delineated by the dotted lines. Two-way arrows indicate integumentary tapetum for width quantification. IT, integumentary tapetum. Scale bars: 50 μm (first and third column); 10 μm (second and fourth column). **b** Statistical data on the width of the integumentary tapetum in 4-DAP seeds from the WT, *atg5-1*, *atg5-4*, and *proATG5:ATG5-GFP* complementation plants are presented in box-and-whisker plots. Data are from 30 independent seeds (n = 30). (Two-tailed Student´s *t* test, ns, no significant difference, $P > 0.05$; ****$P < 0.0001$).

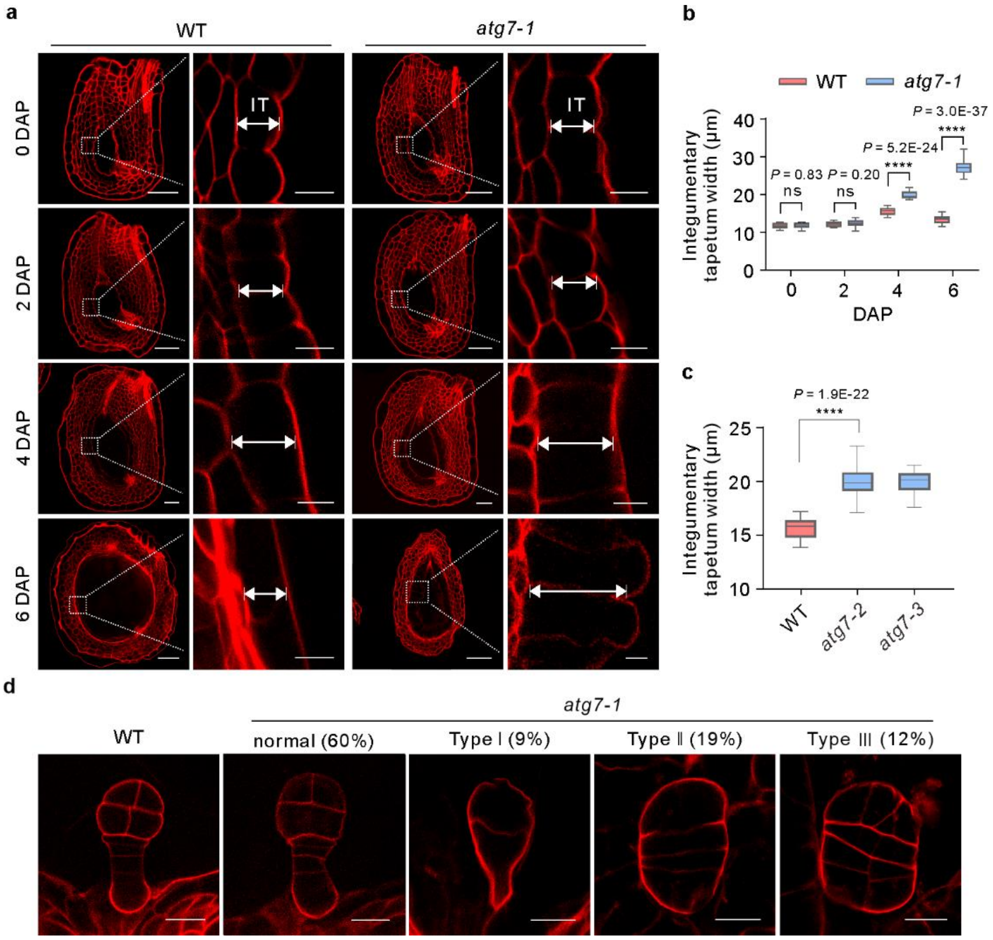

Supplementary Fig. 6 Mutation in *ATG7* results in the defects in the degradation of the integumentary tapetum as well as to embryo pattern formation.

a mPS-PI staining showing the integumentary tapetum in the ovules and seeds of both the WT and *atg7-1* mutant. The right-hand images display enlarged areas delineated by dotted lines. Two-way arrows indicate integumentary tapetum for width quantification. IT, integumentary tapetum; DAP, days after pollination. Scale bars: 50 μm (left) and 10 μm (right) for 0-DAP ovules, 2-DAP seeds, and 4-DAP seeds; 100 μm (left) and 10 μm (right) for 6-DAP seeds. **b,c** Statistical analysis of the width of integumentary tapetum in the WT and *atg7* mutant. The data of integumentary tapetum width are displayed in box-and-whisker plots. Statistical data on integumentary tapetum width in **c** are from 4-DAP seeds. The data were collected from 30 independent seeds (n = 30). **d** Abnormal embryo division patterns were observed in *atg7-1* mutant. The embryos of *atg7-1* mutant could be classified into four distinct types based on their embryonic cell division patterns (n = 100 embryos). Scale bars: 20 μm. (Two-tailed Student´s *t* test, ns, no significant difference, $P > 0.05$; ****$P < 0.0001$).

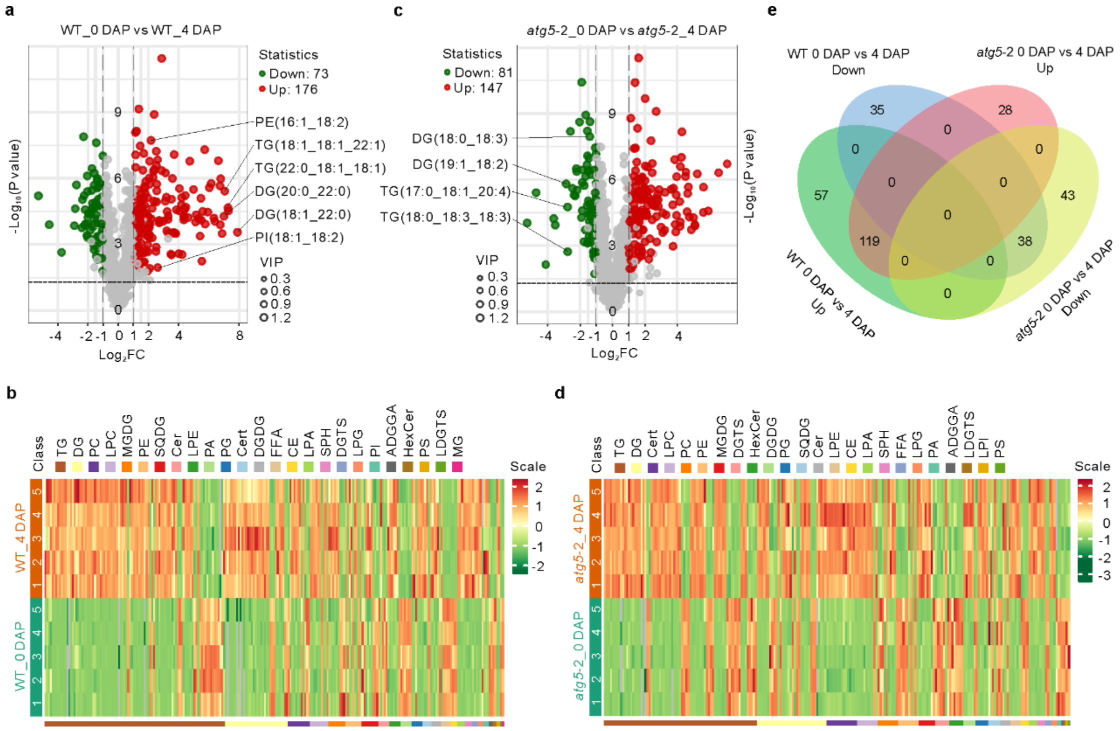

Supplementary Fig. 7 The impact of autophagy deficiency on lipid metabolism during seed development.

a Volcano plot of differential metabolites between WT ovules and 4-DAP seeds. Significantly up-regulated metabolites are shown as red dots and significantly down-regulated metabolites are shown as green dots, with the size of the dots representing VIP values. VIP, variable importance in projection. FC, fold change. n = 5 biologically independent samples. **b** Clustering heat maps showing the relative lipid contents between WT ovules and 4-DAP seeds. **c** Volcano plot of differential metabolites between *atg5-2* ovules and 4-DAP seeds. n = 5 biologically independent samples. **d** Clustering heat maps showing the relative lipid contents between ovules and 4-DAP seeds from *atg5-2* mutant. **e** Venn diagram comparing differential metabolites between WT ovules and 4-DAP seeds, and *atg5-2* ovules and 4-DAP seeds. TG, triacylglycerol; DG, diacylglycerol; MG, monoglyceride; FFA, free fatty acid; PC, phosphatidylcholine; PE, phosphatidylethanolamine; PI, phosphatidylinositol; PA, phosphatidic acid; PS, phosphatidyl serine; PG, phosphatidyl glycerol; LPC, lyso-PC; LPE, lyso-PE; LPI, lyso-PI; LPA, lyso-PA; LPG, lyso-PG; LDGTS, lyso-DGTS; CE, cholesteryl ester; Cer, ceramide; Cert, phytoceramide; HexCer, hexosylceramide; MGDG, monogalactosyl diacylglycerol; PMeOH, phosphatidylmethanol; DGDG, digalactosyl diacylglycerol; SPH, sphingosine; DGTS, diacylglyceryltrimethylhomoserine; SQDG, sulfoquinovosyl diacylglycerol; CoQ, coenzyme Q.

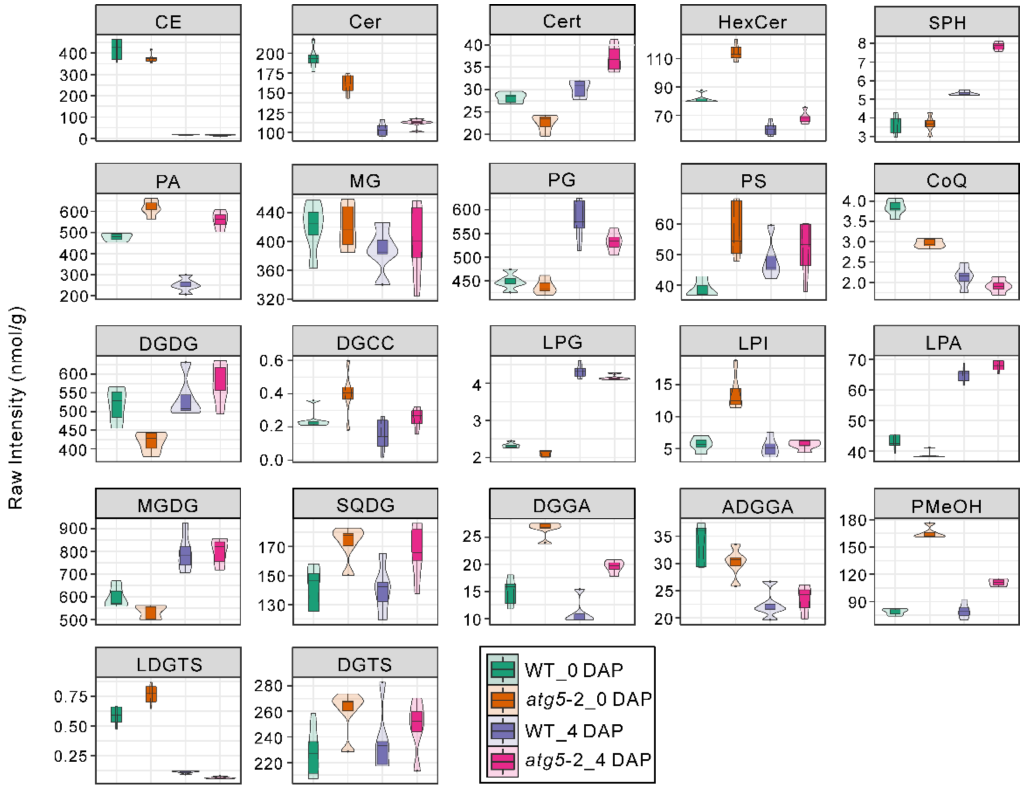

Supplementary Fig. 8 Violin plots displaying the content of various lipid subclasses from the WT and *atg5-2* plants.

The black horizontal line right in the middle represents the median, the middle box represents the quartile range, the thin black line extending from it represents the 95% confidence interval, and the outer shape represents the distribution density of the data. n = 5 biologically independent samples.

Supplementary Table 1. Primers used in this study.

	Primer name	Sequences (5'-3')	Experiment
ATG5 promoter	pATG5-F	TAAAATCGAGTTCAAAATTCTTCTGC	Cloning
	pATG5-R	TATTATCTTCTCTTCTCACCTCTCT	
ATG7 promoter	pATG7-F	GATTTAATCACTTGAAACAGTGTCG	
	pATG7-R	GAAAAAAAATCTTGCTTTATTGGAA	
TPE8 promoter	pTPE8-F	CGAATGAACATGCCCATCCTGGCTT	
	pTPE8-R	TTTTCACACGCTAAAAATAATTACT	
ATG8 CDS	ATG8d-cds-F	ATGGCAAAGAGTTCATTCAAGCAAG	
	ATG8d-cds-R	TTACACCAAGTTGAGGTCGCCA	
atg5 mutant genome	ATG5-geno-F	TCGACTCTTGGCTTTCTCTTTGTAG	PCR and sequencing
	ATG5-geno-R	GCATTGGCACATTCAGAAACTTAC	
atg7 mutant genome	ATG7-geno-F	CATAGAAGAGAACAGCAGCGGTACT	
	ATG7-geno-R	GCCATCCAGGATTACTTGGAAGATG	
ATG5 CRISPR/Cas9 vector construction	UATG5-1	TTTGAGAGGCAAGCCTTTGtgaccaatgttgctccc	Cloning of sgRNA expression cassettes
	gRATG5-1	CAAAGGCTTGCCTCTCAAAgttttagagctagaaat	
	UATG5-2	AGGACGTTCAGGTTCTGCAcaatcactacttcgtct	
	gRATG5-2	TGCAGAACCTGAACGTCCTgttttagagctagaaat	
	UATG5-3	TCCCATACGTATTTCTGTGCtgaccaatggtgctttg	
	gRATG5-3	GCACAGAAATACGTATGGGAgttttagagctagaaat	
ATG7 CRISPR/Cas9 vector construction	UATG7-1	AAGCAGGGAACGCAAACCAAtgaccaatgttgctccc	
	gRATG7-1	TTGGTTTGCGTTCCCTGCTTgttttagagctagaaat	
	UATG7-2	GAGTTGCAGGAGGATCAAGcaatcactacttcgtct	
	gRATG7-2	CTTGATCCTCCTGCAACTCgttttagagctagaaat	
	UATG7-3	ACTCCCTTAGATGCCTGAGAtgaccaatggtgctttg	
	gRATG7-3	TCTCAGGCATCTAAGGGAGTgttttagagctagaaat	
CRISPR/Cas9 vector construction	U-F	CTCCGTTTTACCTGTGGAATCG	Coning of sgRNA expression cassettes
	gRNA-R	CGGAGGAAAATTCCATCCAC	
	Pps-GGL	TTCAGAggtctcTctcgACTAGTATGGAATCGGCAGCAAAGG	
	Pgs-GG2	AGCGTGggtctcGtcagggTCCATCCACTCCAAGCTC	
	Pps-GG2	TTCAGAggtctcTctgacacTGGAATCGGCAGCAAAGG	
	Pgs-GG3	AGCGTGggtctcGtcttcacTCCATCCACTCCAAGCTC	
	Pps-GG3	TTCAGAggtctcTaagacttTGGAATCGGCAGCAAAGG	
	Pgs-GGR	AGCGTGggtctcGaccgACGCGTATCCATCCACTCCAAGCTC	
	PB-L	GCGCGCgGTctcGCTCGACTAGTATGG	PCR
	PB-R	GCGCGCggtctcTACCGACGCGTATCC	
	SP-L	GTCGTGCTCCACATGTTGACCG	Sequencing
	SP-R	CCGACATAGATGCAATAACTTC	
