# Supplementary material for: Autophagy-mediated degradation of integumentary tapetum is critical for embryo pattern formation
Source: Nat Commun. 2024 Mar 27;15:2676. doi: 10.1038/s41467-024-46902-8 (PMC10973531; doi:10.1038/s41467-024-46902-8)
Supplement: Supplementary file 3 — Description of Additional Supplementary Files [file 41467_2024_46902_MOESM3_ESM.pdf]

Description of Additional Supplementary Files:

Supplementary Data 1: List of differentially expressed genes between atg5-2/WT and atg5- 2/atg5-2 proTPE8:ATG5-GFP.

Supplementary Data 2: The resource of lipidomics data.

Supplementary Movie 1: A representative movie displaying a seed from top to bottom sections.

Supplementary Movie 2: A representative movie showing the structure of a seed.
