# Supplementary material for: Autophagy-mediated degradation of integumentary tapetum is critical for embryo pattern formation
Source: Nat Commun. 2024 Mar 27;15:2676. doi: 10.1038/s41467-024-46902-8 (PMC10973531; doi:10.1038/s41467-024-46902-8)
Supplement: Supplementary file 8 — Reporting Summary [file 41467_2024_46902_MOESM8_ESM.pdf]

nature portfolio

Corresponding author(s): Peng Zhao

Last updated by author(s): Feb 26, 2024

Reporting Summary

Nature Portfolio wishes to improve the reproducibility of the work that we publish. This form provides structure for consistency and transparency in reporting. For further information on Nature Portfolio policies, see our Editorial Policies and the Editorial Policy Checklist.

Statistics

For all statistical analyses, confirm that the following items are present in the figure legend, table legend, main text, or Methods section.

n/a	Confirmed	
☐	☒	The exact sample size (*n*) for each experimental group/condition, given as a discrete number and unit of measurement
☐	☒	A statement on whether measurements were taken from distinct samples or whether the same sample was measured repeatedly
☐	☒	The statistical test(s) used AND whether they are one- or two-sided<br>*Only common tests should be described solely by name; describe more complex techniques in the Methods section.*
☒	☐	A description of all covariates tested
☐	☒	A description of any assumptions or corrections, such as tests of normality and adjustment for multiple comparisons
☐	☒	A full description of the statistical parameters including central tendency (e.g. means) or other basic estimates (e.g. regression coefficient) AND variation (e.g. standard deviation) or associated estimates of uncertainty (e.g. confidence intervals)
☐	☒	For null hypothesis testing, the test statistic (e.g. *F*, *t*, *r*) with confidence intervals, effect sizes, degrees of freedom and *P* value noted<br>*Give P values as exact values whenever suitable.*
☒	☐	For Bayesian analysis, information on the choice of priors and Markov chain Monte Carlo settings
☒	☐	For hierarchical and complex designs, identification of the appropriate level for tests and full reporting of outcomes
☐	☒	Estimates of effect sizes (e.g. Cohen's *d*, Pearson's *r*), indicating how they were calculated

Our web collection on statistics for biologists contains articles on many of the points above.

Software and code

Policy information about availability of computer code

Data collection	Fluorescence images were collected by Leica TCS SP8. Images of seeds were collected by Nikon stereomicroscope SMZ25. Cellular ultrastructure was observed with transmission electron microscopy (JEM-1400, JEOL) and imaged with the camera system DS-L1 (Gatan). Lipidomic data was acquired with LC-ESI-MS/MS system (UPLC, ExionLC AD; MS, QTRAP® 6500+ System) , equipped with an ESI Turbo Ion-Spray interface controlled by Analyst 1.6.3 software. RNA-seq data were generated on an MGI DNBSEQ-T7 platform with a 2×150 bp paired-end model.
Data analysis	GraphPad Prism 9 was used for statistical analysis. ImageJ (1.53k) was used for LDs' area calculation and number counting. Unsupervised principal component analysis (PCA) was performed by statistics function prcomp within R (www.r-project.org). R base package (3.5.0) and pheatmap R (1.0.12) were used for lipidomic analysis. STAR (v.2.7.11a), RSEM (v.1.3.3), DESeq2, ClusterGVis package and R package ggplot2 (v.3.4.3) were used for RNA-seq data analysis.

For manuscripts utilizing custom algorithms or software that are central to the research but not yet described in published literature, software must be made available to editors and reviewers. We strongly encourage code deposition in a community repository (e.g. GitHub). See the Nature Portfolio guidelines for submitting code & software for further information.

Data

Policy information about availability of data

All manuscripts must include a data availability statement. This statement should provide the following information, where applicable:

- Accession codes, unique identifiers, or web links for publicly available datasets
- A description of any restrictions on data availability
- For clinical datasets or third party data, please ensure that the statement adheres to our policy

Raw data of lipidomics are provided with this paper. RNA-seq data have been uploaded to the NCBI Gene Expression Omnibus (GEO) under accession GSE248624 (https://www.ncbi.nlm.nih.gov/geo/query/acc.cgi?acc=GSE248624). Source data are provided with this paper. All seeds and other materials related to the findings of this study are available from the corresponding authors upon reasonable request.

Research involving human participants, their data, or biological material

Policy information about studies with human participants or human data. See also policy information about sex, gender (identity/presentation), and sexual orientation and race, ethnicity and racism.

Reporting on sex and gender	n/a
Reporting on race, ethnicity, or other socially relevant groupings	n/a
Population characteristics	n/a
Recruitment	n/a
Ethics oversight	n/a

Note that full information on the approval of the study protocol must also be provided in the manuscript.

Field-specific reporting

Please select the one below that is the best fit for your research. If you are not sure, read the appropriate sections before making your selection.

☒ Life sciences ☐ Behavioural & social sciences ☐ Ecological, evolutionary & environmental sciences

For a reference copy of the document with all sections, see nature.com/documents/nr-reporting-summary-flat.pdf

Life sciences study design

All studies must disclose on these points even when the disclosure is negative.

Sample size	The sample size for each experiment was determined based on our prior experience performing similar researches, as well as previously published papers. The sample sizes for all experiments are reported in the manuscript.
Data exclusions	No data was excluded.
Replication	All experiments were performed with at least two (mostly three) independent replicates.
Randomization	Although typical randomization was not necessary for the experiments in the present study, we randomly selected plant materials with the same genotype for the studies.
Blinding	Typical blinding was not necessary for the experiments in the present study. To increase the reliability of the findings, all experiments were repeated a minimum of two times, and the ovules and seeds for phenotype analysis were randomly collected from the different plants.

Reporting for specific materials, systems and methods

We require information from authors about some types of materials, experimental systems and methods used in many studies. Here, indicate whether each material, system or method listed is relevant to your study. If you are not sure if a list item applies to your research, read the appropriate section before selecting a response.

Materials & experimental systems

n/a	Involved in the study
☐	☒ Antibodies
☒	☐ Eukaryotic cell lines
☒	☐ Palaeontology and archaeology
☒	☐ Animals and other organisms
☒	☐ Clinical data
☒	☐ Dual use research of concern
☐	☒ Plants

Methods

n/a	Involved in the study
☒	☐ ChIP-seq
☒	☐ Flow cytometry
☒	☐ MRI-based neuroimaging

Antibodies

Antibodies used

Anti-ATG8A, Abcam, ab77003, 1:100 dilution for immunofluorescence; 1:1000 dilution for western blot.
Anti-ATG5, Abclonal, E3631; 1:2000 dilution
Anti-ATG7, Abclonal, E3633; 1:100 dilution for immunofluorescence; 1:2000 dilution for western blot.
Plant Actin, Abbkine, A01050, 1:2000 dilution
GFP–Tag Mouse mAb, DIA-AN, 2057; 1:100 dilution
HRP Goat Anti-Mouse IgG, Abclonal, AS003; 1:4000 dilution
HRP Goat Anti-Rabbit IgG, Abclonal, AS014; 1:4000 dilution
Goat Anti-Rabbit IgG H&L (Alexa Fluor® 488), Abcam, ab150077, 1:200 dilution.
Goat Anti-Rabbit IgG (H+L) (Alexa Fluor™ 594), Thermo Fisher Scientific, A-11012,1:200 dilution
Goat Anti-Mouse IgG (H+L) (Alexa Fluor™ 488), Thermo Fisher Scientific, A-11001; 1:200 dilution

Validation

Following antibodies are validated by the manufacturer.
https://www.abcam.cn/products/primary-antibodies/apg8aatg8a-antibody-ab77003.html
https://abbkine.com/datasheet/A01050.pdf
https://abclonal.com.cn/catalog/AS003
https://abclonal.com.cn/catalog/AS014
https://dia-an.com/index/product.html?pro_id=4453&aid=48,49#view_zjy
https://www.abcam.cn/products/secondary-antibodies/goat-rabbit-igg-hl-alexa-fluor-488-ab150077.html
https://www.thermofisher.cn/cn/zh/antibody/product/Goat-anti-Rabbit-IgG-H-L-Cross-Adsorbed-Secondary-Antibody-Polyclonal/A-11012
https://www.thermofisher.cn/cn/zh/antibody/product/Goat-anti-Mouse-IgG-H-L-Cross-Adsorbed-Secondary-Antibody-Polyclonal/A-11001

Dual use research of concern

Policy information about dual use research of concern

Hazards

Could the accidental, deliberate or reckless misuse of agents or technologies generated in the work, or the application of information presented in the manuscript, pose a threat to:

No	Yes	
[x]	[ ]	Public health
[x]	[ ]	National security
[x]	[ ]	Crops and/or livestock
[x]	[ ]	Ecosystems
[x]	[ ]	Any other significant area

Experiments of concern

Does the work involve any of these experiments of concern:

No	Yes	
[x]	[ ]	Demonstrate how to render a vaccine ineffective
[x]	[ ]	Confer resistance to therapeutically useful antibiotics or antiviral agents
[x]	[ ]	Enhance the virulence of a pathogen or render a nonpathogen virulent
[x]	[ ]	Increase transmissibility of a pathogen
[x]	[ ]	Alter the host range of a pathogen
[x]	[ ]	Enable evasion of diagnostic/detection modalities
[x]	[ ]	Enable the weaponization of a biological agent or toxin
[x]	[ ]	Any other potentially harmful combination of experiments and agents

Plants

Seed stocks	Nicotiana tabacum L. cv. Petite Havana SR1 (Hubei Academy of Agricultural Sciences)
Novel plant genotypes	proATG5:H2B-GFP, proATG7:H2B-GFP, proTPE8:ATG5-GFP, proTPE8:GFP-ATG8, and proATG5:ATG5-GFP transgenic plants were generated using the Agrobacterium tumefaciens-mediated transformation method. For the analysis, we used at least two independent T2 transgenic lines. The atg5 and atg7 mutants were generated using the CRISPR/Cas9-mediated genome editing system, with the target sequences "GCACAGAAATACGTATGGGA" for ATG5 and "TCTCAGGCATCTAAGGGAGTG" for ATG7. Three independent mutant T2 lines of ATG5 and ATG7 were used for phenotype analysis.
Authentication	The mutations in ATG5 and ATG7 genome sequences were determined by Sanger sequencing on PCR products that covered the target sites. Protein levels of ATG5 and ATG7 in their respective atg mutants were evaluated through western blot. To exclude potential off-target effects, three independent atg lines were used for phenotype analysis. Moreover, genetic complement experiments were performed to further confirm the role of autophagy in early seed development.
